# Tetrahydrouridine Inhibits Cell Proliferation through Cell Cycle Regulation Regardless of Cytidine Deaminase Expression Levels

**DOI:** 10.1371/journal.pone.0037424

**Published:** 2012-05-16

**Authors:** Naotake Funamizu, Curtis Ray Lacy, Kaori Fujita, Kenei Furukawa, Takeyuki Misawa, Katsuhiko Yanaga, Yoshinobu Manome

**Affiliations:** 1 Department of Molecular Cell Biology, Institute of DNA Medicine, The Jikei University School of Medicine, Tokyo, Japan; 2 University of California Irvine, Irvine, California, United States of America; 3 Department of Cell Growth and Differentiation, Center for iPS Cell Research and Application, Kyoto University, Kyoto, Japan; 4 Department of Surgery, The Jikei University School of Medicine, Tokyo, Japan; Institute of Clinical Physiology, c/o Toscana Life Sciences Foundation, Italy

## Abstract

Tetrahydrouridine (THU) is a well characterized and potent inhibitor of cytidine deaminase (CDA). Highly expressed CDA catalyzes and inactivates cytidine analogues, ultimately contributing to increased gemcitabine resistance. Therefore, a combination therapy of THU and gemcitabine is considered to be a potential and promising treatment for tumors with highly expressed CDA. In this study, we found that THU has an alternative mechanism for inhibiting cell growth which is independent of CDA expression. Three different carcinoma cell lines (MIAPaCa-2, H441, and H1299) exhibited decreased cell proliferation after sole administration of THU, while being unaffected by knocking down CDA. To investigate the mechanism of THU-induced cell growth inhibition, cell cycle analysis using flow cytometry was performed. This analysis revealed that THU caused an increased rate of G1-phase occurrence while S-phase occurrence was diminished. Similarly, Ki-67 staining further supported that THU reduces cell proliferation. We also found that THU regulates cell cycle progression at the G1/S checkpoint by suppressing E2F1. As a result, a combination regimen of THU and gemcitabine might be a more effective therapy than previously believed for pancreatic carcinoma since THU works as a CDA inhibitor, as well as an inhibitor of cell growth in some types of pancreatic carcinoma cells.

## Introduction

Pancreatic carcinoma is the fourth leading cause of cancer-related deaths in the United States. In 2010, the number of newly diagnosed cases of pancreatic carcinoma was estimated to be 43,140 with 36,800 deaths resulting from related complications [1]. Unfortunately, a dismal 20% of all patients are candidates for surgical resection [2], while unresectable cases generally receive chemotherapy comprised of a standard gemcitabine regimen (2′,2′-difluorocytidine). Despite gemcitabine's effectiveness, almost all patients have an advanced stage disease that is inherently resistant or acquires resistance to gemcitabine [3]–[5]. The vexing aspects of the disease support the urgent need for further exploration into the mechanisms conferring this resistance. A better understanding of these mechanisms will play an important role in overcoming gemcitabine resistance and help combat a bleak survival rate. Some studies have shown that gemcitabine resistance is associated with cytidine deaminase (CDA) [6]–[8]. CDA is the catabolic enzyme of gemcitabine which eventually transforms it to an inactive metabolite (2′,2′-difluorodeoxyuridine). Tetrahydrouridine (THU), a well known and potent inhibitor of CDA, competitively blocks the enzyme's active site more effectively than intrinsic cytidine [9], [10]. Previous reports suggest that a combination therapy of gemcitabine and THU is more effective for several types of cancer [11]–[13] due to the maintenance of gemcitabine's half-life [14]–[16]. In this study, CDA expression was measured in three different pancreatic carcinoma cell lines (Panc-1, MIAPaCa-2, and BxPC-3). CDA was expressed higher in BxPC-3 compared to both Panc-1 and MIAPaCa-2. THU was used in a cytotoxic assay to increase gemcitabine sensitivity. Unexpectedly, MIAPaCa-2 cells showed increased gemcitabine sensitivity upon THU exposure despite having a 1,000-fold lower level of CDA mRNA compared to BxPC-3. Additionally, we found that MIAPaCa-2 cell growth was inhibited by sole administration of THU. To further demonstrate this relationship, we confirmed THU's function as a cell growth inhibitor in lung carcinoma cell lines. Our results showed that THU suppressed cell growth in H441 and H1299 cell lines. We then investigated the mechanism of THU-induced cell growth inhibition in both pancreatic and lung carcinoma cell lines, and hypothesized that THU could be an independent inhibitor of tumor cell proliferation in spite of being known as a harmless drug to humans.

## Materials and Methods

### Drugs and chemicals

Gemcitabine (difluorodeoxycytidine, dFdC) was kindly gifted by Eli Lilly (Indianapolis, IN, USA). Tetrahydrouridine was purchased from Calbiochem (La Jolla, CA, USA) as a sterile white powder and stored at −20°C. Both drugs were dissolved in sterile distilled water and diluted in culture medium before being used.

### Cell lines

The following human pancreatic and lung carcinoma cell lines were used in this study: Panc-1, MIAPaCa-2, BxPC-3, H322, H441, and H1299. All cell lines were obtained from the American Type Culture Collection (Rockville, MD). Panc-1 and MIAPaCa-2 cells were cultured in Dulbecco's Modified Eagle Medium (DMEM) supplemented with 10% fetal bovine serum (FBS) and antibiotics (100 µg/ml penicillin and 100 µg/ml streptomycin). BxPC-3, H322, H441, and H1299 cells were cultured in RPMI 1640 with the same supplements. All cell lines were routinely passaged as monolayers at 37°C in a humidified atmosphere of 95% air and 5% CO2.

### RNA preparation and RT-PCR (Real-Time Quantitative PCR)

Total RNA was extracted from cultured cells using Trizol (Invitrogen: Tokyo, Japan) according to the manufacturer's recommendations. Cell pellets were suspended in a 1 ml aliquot of Trizol per one well of a 6-well plate. 6 µg of the isolated RNA was used for reverse transcription (GE Health Care, Buckinghamshire, UK) utilizing random primers (6 mer) according to the manufacturer's protocol. cDNA samples were diluted and stored at −20°C until needed. Gene expression levels were measured with Taqman real-time polymerase-chain reaction (Applied Biosystems: Foster City, CA) containing custom-designed probes for 2 genes (CDA, ID: Hs00156401_m1; and E2F1, ID: Hs00174164_m1) and GAPDH (ID: Hs99999901_s1) as an internal control. The expression levels of respective cells were analyzed using the relative quantification (ΔΔ Ct) method. Each sample was assayed in triplicate.

### Gemcitabine chemo-sensitivity with tetrahydrouridine (THU)

A drug sensitivity assay was performed essentially as described in a previous report [11]. Cells were seeded in 96-well plates at 2,500∼5,000 cells/well in triplicate. After incubating for 12 hrs, cell viability was determined by treating cells with stepwise 4-fold serial dilutions of gemcitabine (from 100 µM), and incubating at 37°C for 96 hrs with or without THU. To evaluate cell viability, all cells were fixed with 25% glutaraldehyde for 30 min at room temperature and then stained with 200 µl of 0.05% methylene blue for 20 min. The dye was eluted with 0.33 M HCl for 20 min with agitation. Absorbance was measured in a microplate reader (model 3550, Bio-Rad, Tokyo, Japan) at 598 nm. The 50% inhibitory concentration for cell growth (IC50) was calculated.

### Cell growth assay

Cell growth for pancreatic and lung carcinoma cell lines was carried out using the colorimetric methylene blue assay in 96-well plates at a density of 5,000 cells/well. Cells were either exposed or not exposed to THU, counting the first 12 hrs as Day 0. Mean values were calculated from three different wells in triplicates for four days.

### Cell cycle analysis by flow cytometry

For cell cycle analysis by flow cytometry, cells were seeded in a 10 cm dish at 60% confluency. After 12 hrs, 100 µM THU was added to each dish for three days. Cells were then dissociated using trypsin-EDTA (trypsin 0.25%; EDTA 0.02%) for 2 to 5 min at 37°C, transferred to a 15 mL tube, and washed three times with PBS. Cells were then fixed overnight in 70% ethanol at 4°C before being washed again with PBS and resuspended in PBS-triton-X100 (0.1%). The cells were concomitantly treated with RNaseA (Sigma Aldrich, San Luis, MO) and stained with propidium iodide. Cell cycle status was determined using a FACS calibur flow cytometer (Becton Dickinson, Oxford, UK) and analyzed using FlowJo-887 software.

### Ki-67 staining

To perform Immunocytochemistry (ICC), cells were seeded in chamber slides at 20–30% confluency. After 12 hrs of incubation, 100 µM tetrahydrouridine (THU) was added for four days. The ICC procedures were performed after fixing the cell samples with 4% paraformaldehyde in PBS for 15 min. For permeabilization, samples were incubated for 10 min with PBS containing 0.3% Triton X-100. Cells were then treated with a blocking solution containing 1% BSA in PBS for 60 min. Samples were incubated for 90 min with the Ki-67 antibody (Abcam, Tokyo, Japan) in blocking buffer before applying the secondary goat anti-rabbit antibody for 60 min. Slides were treated with the streptavidin–biotin complex reagent (Dako, Glostrup, Denmark) for 30 min and developed with 0.4% diaminobenzidine (DAB)/H_2_O_2_. All stages of the staining process were performed at room temperature and the slides were washed in PBS. The slides were mounted and analyzed using light microscopy after stopping the DAB reaction. The evaluation of ICC staining was performed by counting all positive reactions in 1000 cells at a magnification of 400× in the whole cell area.

### Transient transfections of cytidine deaminase -siRNA

To knockdown CDA expression in MIAPaCa-2, H441, and H1299 cells, all cells were seeded at 70–80% confluency in 6-well plates. Two cytidine deaminase and a negative control siRNAs were purchased from Invitrogen (Tokyo, Japan). All siRNAs were individually transfected in each cell line at a final concentration of 60 nM per well with Lipofectamine 2000 (Invitrogen, Tokyo, Japan). To measure mRNA levels, cells were incubated for 48 hrs and then harvested for real-time PCR analysis. 12 hrs after transfecting, cell growth was determined following the procedure outline above. We also tested whether CDA inhibition plus THU treatment surpasses the efficiency of THU alone using a proliferation assay. Transfected cells were seeded in a 96-well plate overnight and THU was added at 100 µM. As prescribed above, the OD was measured at Day0, 2 and 4.

### Immunoblotting analysis

In order to evaluate whether tetrahydrouridine (THU) affects the cell cycle related factor at the protein level, western blotting was performed. Four days after adding 100 µM THU or PBS as a control, protein was extracted from the cell pellet using RIPA buffer with protease inhibitor (Invitrogen: Tokyo, Japan). Protein concentration was measured using Bio-Rad protein assay solution. Equal amounts of total protein were separated using SDS-PAGE gels under reducing conditions. The protein was then transferred to a polyvinylidene difluoride (PVDF) membrane (Invitrogen, Tokyo, Japan) before being blocked with 5% non-fat milk in TBS-Tween. The membrane was then incubated with primary antibodies against E2F1 (Santa Cruz, Santa Cruz CA) and β-actin (Cell Signaling Technology, Tokyo, Japan) at a dilution of 1∶1,000. The appropriate primary antibodies were followed by horseradish peroxidase (HRP)-conjugated secondary antibodies (Amersham Pharmacia Biotech, Piscataway, NJ) at a dilution of 1∶5,000. Visualization was achieved using SuperSignal West chemiluminescent solution (Pierce, Rockford, IL).

### Statistical analyses

All results were conducted in triplicate and carried out on at least two occasions. Graphpad Prism v5.0 (Graphpad Software Inc, La Jolla, CA) was used for statistical analysis. Levels of significance for comparison between all cell lines were determined by the Student's *t*-test distribution or ANOVA analysis. A two-sided value of <0.05 was considered significant.

## Results

### Expression of cytidine deaminase (CDA) using real-time PCR

**Figure 1:**
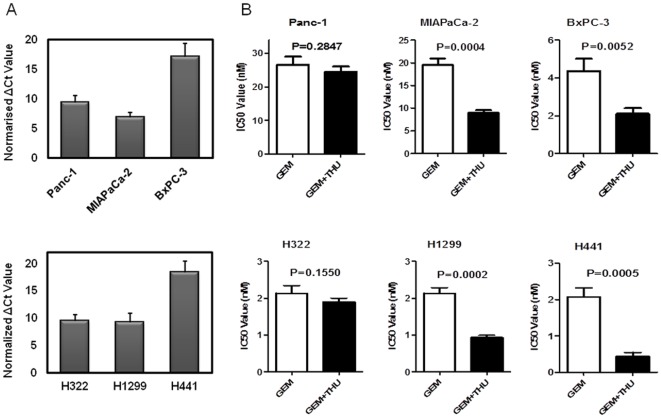
Inhibition of cytidine deaminase (CDA) contributes to improved gemcitabine sensitivity. **A.** Levels of CDA mRNA in pancreatic and lung carcinoma cell lines were evaluated by real-time PCR for Panc-1, MIAPaCa-2, BxPC-3, H322, H1299, and H441. Normalized δCt was calculated as 35-(CDA Ct-GAPDH Ct). **B. **Three pancreatic and three lung cancer cell lines were cultured with just gemcitabine (GEM) alone, or with GEM and 100 mM tetrahydrouridine (THU). IC50 was determined using a colorimetric assay as described in the experimental procedures. Values are means ±SD, and results are representative of 3 independent experiments.

mRNA expression of CDA was examined by RT-PCR in all cell lines. The CDA mRNA level in BxPC-3 was higher than in both Panc-1 and MIAPaCa-2 cells. H441 had a higher expression level of CDA than H322 and H1299 (Fig. 1A). All samples were regarded as evaluable on the basis of GAPDH Ct levels.

### Combination therapy with gemcitabine and tetrahydrouridine (THU) improves gemcitabine sensitivity in MIAPaCa-2 and H1299 in spite of low cytidine deaminase (CDA) expression

To test how THU affects the gemcitabine-mediated anti-neoplastic effect on pancreatic and lung carcinoma cells, a combination therapy was performed. As expected, high CDA expression in BxPC-3 and H441 resulted in improved gemcitabine sensitivity after a 100 µM THU treatment. The sensitivity of BxPC-3 and H441 cell lines increased by as much as approximately 2.1 and 4.4 fold respectively. On the other hand, MIAPaCa-2 and H1299 cells unexpectedly became more sensitive to gemcitabine with low CDA expression. MIAPaCa-2 and H1299 cells showed a change in IC50 of 2.2 and 2.3 fold respectively. However, Panc-1 and H322 cells did not show significant changes in drug sensitivity (Fig. 1B). These data suggested that THU could sensitize some pancreatic and lung carcinoma cells to gemcitabine-induced cell death regardless of CDA expression levels.

### Tetrahydrouridine (THU) controls cell growth independently

**Figure 2:**
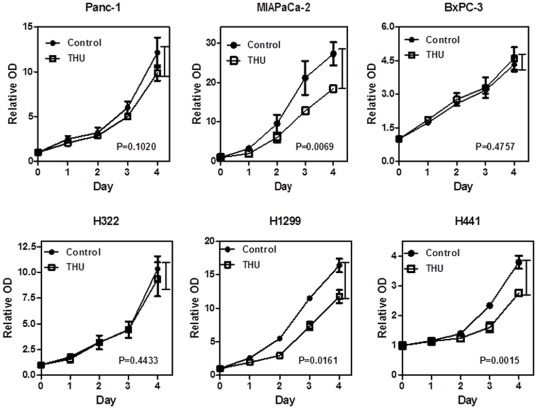
Tetrahydrouridine (THU) inhibits cell growth in MIAPaCa-2, H1299, and H441. Tetrahydrouridine (THU) significantly reduced cell growth by colorimetric assay in MIAPaCa-2, H441, and H1229 cell lines as compared with the respective control. Values are means of three independent experiments performed in triplicate. An error bar is presented as S.D. Day4 data were used for the statistical analysis.

To examine how THU increases gemcitabine sensitivity, even in cells with low levels of CDA expression, a cell proliferation assay was performed. We found that THU independently suppressed tumor proliferation in MIAPaCa-2, H441, and H1299 cells compared to the control (P = 0.0069, 0.0161 and 0.0015 respectively). However, in Panc-1, BxPC-3 and H322 cells, growth inhibition was not indicated (Fig. 2). A t-test was performed using data collected at Day4.

### Tetrahydrouridine (THU) inhibits S-phase without apoptosis

**Figure 3:**
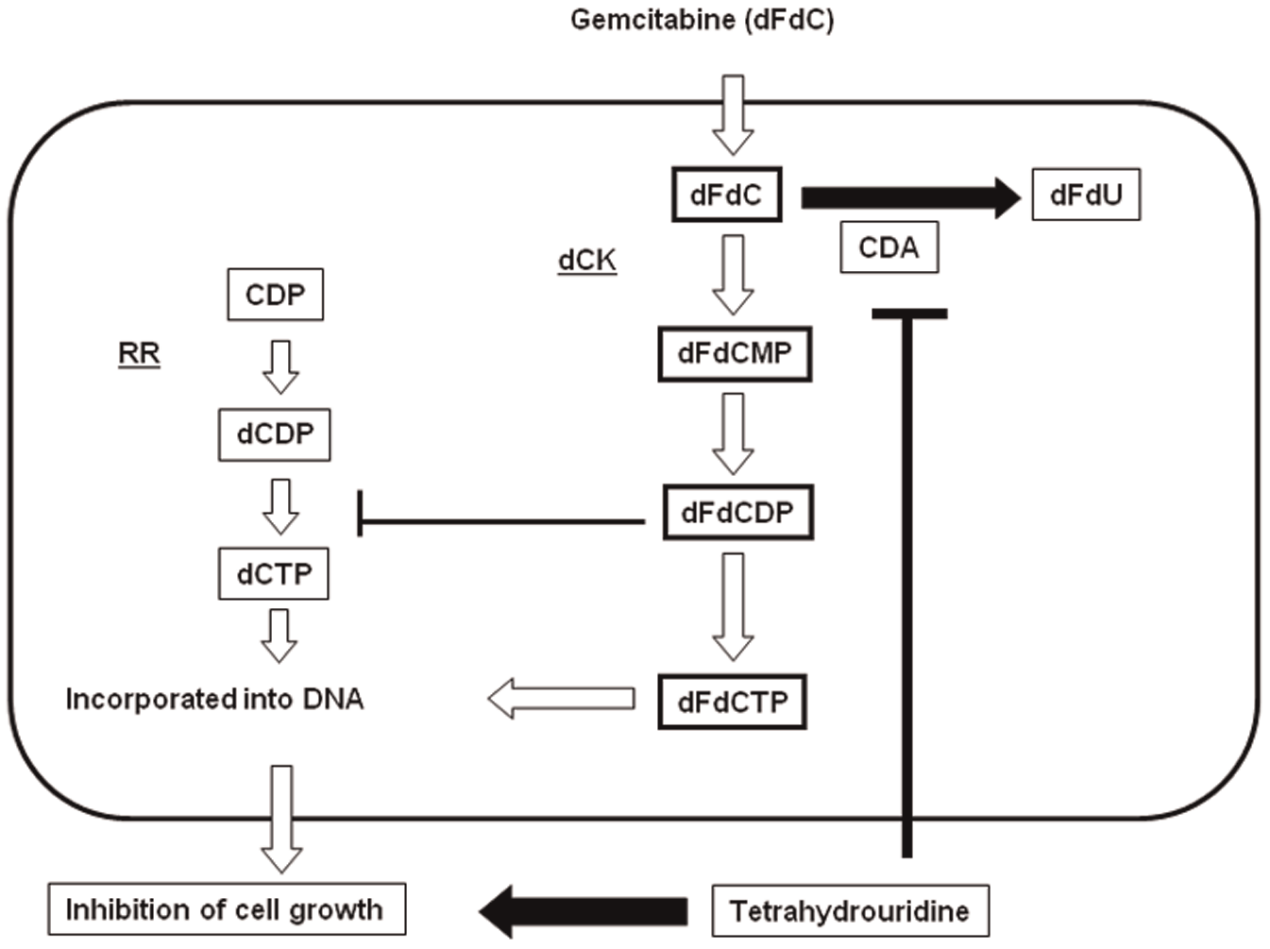
Metabolic pathway of gemcitabine. Gemcitabine (2′,2′-difluorodeoxycytidine, dFdC) is taken in DNA through the active diphosphate (dFdCDP) and triphosphate (dFdCTP) by deoxycytidine kinase (dCK). dFdCTP can inhibit ribonucleotide reductase (RR). On the contrary, Cytidine deaminase (CDA) inactivates dFdC to 2′,2′-difluorodeoxyuridine (dFdU) by deamination. Tetrahydrouridine (THU) prevents dFdC from being inactivated by binding CDA directly. THU also leads to the potential for cell growth inhibition.

**Figure 4:**
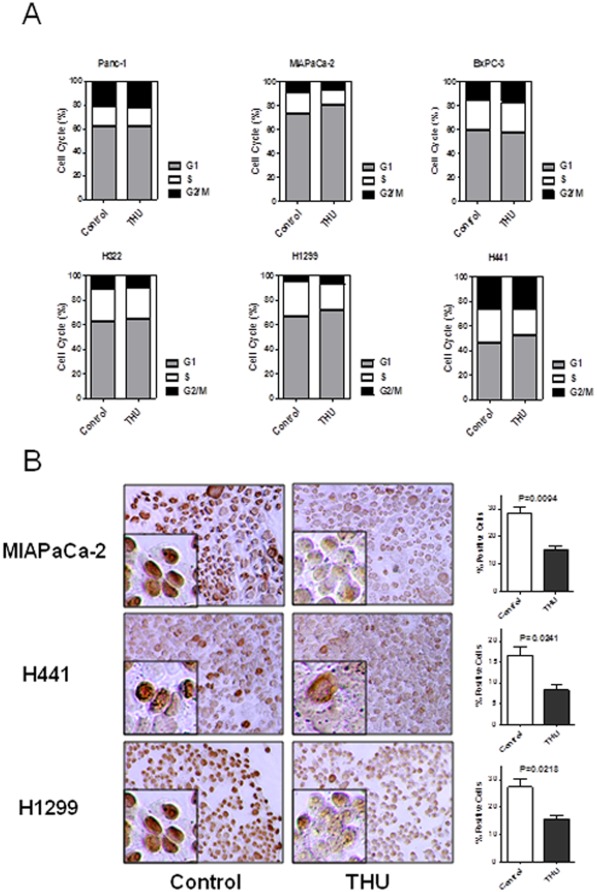
THU (Tetrahydrouridine) influences the cell cycle in MIAPaCa-2, H441 and H1299. **A.** After 3 days of THU exposure, the cell cycle was analyzed using flow cytometry. In accordance with cells whose growth was inhibited by THU, MIAPaCa-2, H441, and H1229 cell lines exhibited an increased rate of the G1-phase and a decrease in the S-phase. **B.** Cells were processed for immuno-cytochemistry with an antibody against Ki-67. Microscopic images of Ki-67-stained cells are shown in THU-treated cells and non-treated cells. Nuclei were stained with DAB. Data represents the mean ± S.D. of 3 independent experiments and significance values relate to comparisons between medium and THU treated cells.

Proliferation data collected from cells treated with THU suggested that THU might have the potential to control cell growth in the absence of CDA inhibition (Fig. 3). To investigate how THU inhibits cell growth, flow cytometry was used. The cell lines which reflected a change in cell growth showed a significant alteration in the rate of S-phase after adding THU. The rate of change in S-phase was 6.0±0.5% (P = 0.0023), 5.8±0.8% (P = 0.0063) and 7.5±1.5% (P = 0.0131) in MIAPaCa-2, H441, and H1299 cells respectively (Fig. 4A). A paired t-test was used for evaluating the difference in the S-phase. Because a sub-G1 curve was not present, we concluded that THU did not induce apoptosis.

### Tetrahydrouridine (THU) regulates Ki-67 antigen

Ki-67 antigen expression was consistently altered in the presence of THU compared to the control in MIAPaCa-2, H441, and H1299 cells. As indicated by positive nuclear staining, proliferation rates were decreased with mean values of 15.0%±1.5%, 8.3%±1.2%, and 15.7%±1.2% respectively compared to 28.3%±2.4%, 16.7%±2.0%, and 27.3%±3.0% in the medium control (Fig. 4B).

### Knock-down of cytidine deaminase (CDA) has no influence on cell proliferation

**Figure 5:**
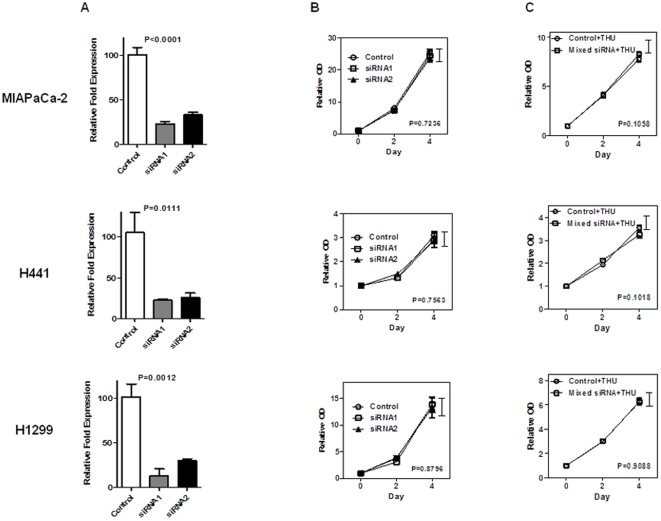
Cytidine deaminase (CDA) inhibition does not participate in cell proliferation. **A.** After transfecting siRNA against CDA, real-time PCR analysis of CDA expression in MIAPaCa-2, H441, and H1299 exhibited cell growth inhibition by THU. Statistical significance was confirmed using a t-test at Day4. **B.** After knocking down CDA, a proliferation assay was performed. Down-regulated CDA did not affect cell growth in MIAPaCa-2, H441, and H1229 cell lines. **C.** A combination therapy of CDA knockdown and THU did not show any differences compared to THU alone.

To examine whether THU affects tumor proliferation through the inhibition of CDA, two different siRNAs against CDA were used in this study. Knockdown efficiency was evaluated using real-time PCR (Fig. 5A). After selecting the most effective siRNA, a cell growth assay was performed as previously described. Cell proliferation was not significantly altered after sufficiently knocking down CDA expression, proving that THU-induced growth inhibition was in a CDA-independent manner (Fig. 5B). Moreover, to investigate whether a combination treatment with CDA inhibition and THU generates a synergistic or additive effect compared to THU alone, a proliferation assay under these conditions was performed. Unfortunately, a difference was not observed in all the cell lines.

### Tetrahydrouridine (THU) controls the G1/S check point through E2F1

**Figure 6:**
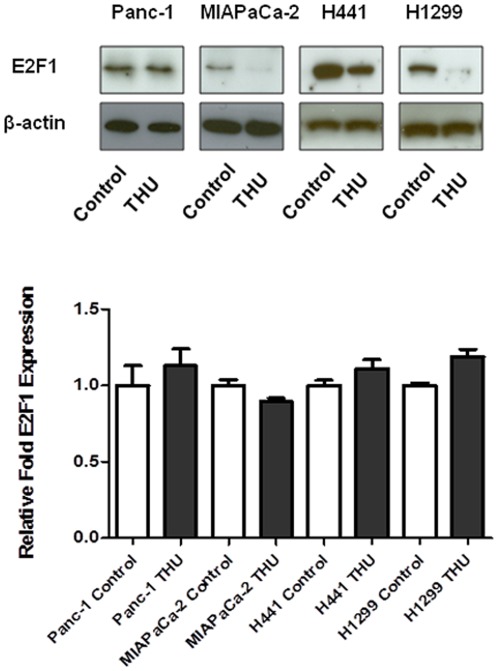
Tetrahydrouridine (THU) inhibits E2F1 at the protein level which is associated with the G1/S transition. Panc-1, MIAPaCa-2, H441, and H1229 cells were treated with THU (100 µM) for 96 hrs as indicated. Subsequently, E2F1 protein levels were analyzed by western blotting. THU could down-regulate E2F1 expression. However, THU did not affect E2F1 mRNA expression level.

On the basis of data compiled by flow cytometry, we examined the G1/S check-point related factor by blotting analysis. In MIAPaCa-2, H441, and H1299 cells, E2F1 expression was decreased after administering THU compared to the control (Fig. 6). In contrast, Panc-1 cells did not show a significant change in intensity. However, other factors including Rb, pRb and cyclin-E/D were at similar levels in both treatment and control cells (Data not shown).

## Discussion

CDA is a prominent enzyme in the catabolism of cytosine nucleoside analogues, and is often over-expressed in cancer cells that have acquired resistance to those analogues [17]–[19]. The processing of drugs by CDA typically results in either a loss of pharmacological activity or the acquisition of undesirable side-effects. Cumulatively, these issues have stimulated researchers' interest in the development of superior CDA inhibitors as therapeutic agents [15], [16], [20], [21]. It has been reported that CDA levels could predict gemcitabine-induced toxicities, as well as negative efficacy [22], [23]. Some evidence suggests that CDA was associated with poor prognosis in acute myeloid leukemia [24]. Conversely, a recent report suggested that the CDA single-nucleotide polymorphism (SNPs), CDA*3 (208 G>A), was significantly associated with a low level of CDA activity, decreased gemcitabine clearance, and drug associated toxicities in Japanese patients [25]. Tibaldi *et al*. also showed that the CDA Lys27Lys (AA) polymorphism was associated with better clinical outcome [26]. Similarly, low plasma CDA activity and heterozygous CDA*3 were also significantly associated with prolonged overall survival in advanced pancreatic carcinoma [27]. Accordingly, down-regulation of CDA plays an important role in deoxycytidine analogues sensitivity and patient prognosis.

Tetrahydrouridine (THU) is a specific inhibitor of cytidine deaminase (CDA) which can suppress deamination in the catabolism of cytotoxic deoxycytidine analogues like ara-C and gemcitabine [28]. During the 1970s, several clinical trials were performed with the combination of THU and Ara-C in patients with acute leukemia [29], [30]. It was reported that THU increased serum Ara-C levels and that the response rates achieved with the combination were comparable to those with Ara-C alone. A phase I study on 5-fluoro-2′-deoxycytidine (FdCyd) and THU was recently conducted in patients with advanced solid tumors to determine its maximum tolerated dose [20]. Even now, several clinical trials exploring the benefits of combining THU and FdCyd are still ongoing with support from the National Institutes of Health (Protocol ID: 09-C-0214, 06-C-0221). CDA is considered not only a predictive marker of chemo-sensitivity to antitumor deoxycytidine analogs, but also as a useful target for biochemical modulation of these analogs. As just described, further THU research is still expected to produce potentially useful results. Selection criteria of patients and THU bioavailability are cited as possible solutions to clinically improve its efficacy. Since several reports showed that a combination therapy including THU is effective against highly expressed CDA in cells [11], [13], patients may be selected according to CDA expression. Also, to maximize the expected advantage of THU, dosage adjustments or the method of administration might be reconsidered. THU stability is also an important problem in utilizing THU as efficiency as possible. It has been suggested that THU bioavailability is approximately no more than 20% [31]. Beumer *et al*. have reported that improved bioavailability has been achieved by delivering THU as a more lipophilic pro-drug with increased THU stability from 20% to 30% [31].

Gemcitabine is currently the gold standard treatment for pancreatic carcinoma despite its efficacy being extremely limited. Hence, a combination regimen including gemcitabine is desirable to enhance therapeutic efficacy. Recently, considerable efforts have been put into the development of molecular targeted drugs based on the inhibition of growth factor receptors and angiogenesis [32]–[34]. A combination therapy using these drugs is more effective than gemcitabine alone. However, clinical benefit still remains insufficient [32]–[34], and high costs along with the risks of side effects of such agents are clinically important. Due to these factors, existing drugs with reliable efficacy and superior cost performance, such as thalidomide and hydroxyurea, need to be reconsidered for tailor-maid therapies [35]–[41].

In the present study, we found that THU could influence a tumor's cell cycle (G1/S phase) in particular cell types. Also, Ki-67 staining supported the possibility that THU inhibits cell proliferation, but did not induce apoptosis judging from the results of flow cytometry and an apoptosis assay (data not shown). A CDA knockdown experiment indicated that CDA inhibition did not influence cell proliferation. To be similar efficacy in MIAPaCa-2 was then verified by carrying out the same assays in lung carcinoma cell lines (H441 and H1299). To examine how THU controls the G1/S transition, we evaluated the G1/S phase related protein after THU treatment. Our data suggested that THU itself could suppress cell growth through the inhibition of E2F1 at the protein level without inducing apoptosis. The E2F1 protein is a member of the E2F family of transcription factors, and is involved in the regulation of cell cycle progression at the G1/S transition. E2F1 activity is tightly controlled in a cell cycle dependent manner by the binding of dephosphorylated retinoblastoma (pRb). In response to mitogenic or non-mitogenic stress signals, after pRb is phosphorylated by cyclin dependent kinase (CDK), released E2F1 can activate cell growth promoting genes in the late G1 phase [42]. We found that THU can control the G1/S transition through the down-regulation of E2F1.

Finally, a second chemotherapy regimen for advanced pancreatic carcinoma is a clinically relevant issue to be resolved urgently. In this study, we demonstrated that THU acts on some tumor cell lines to be both A) independently cytotoxic and B) to sensitize gemcitabine cytotoxicity through E2F1 in both CDA-high and CDA-low expressing cells. Consequently, THU could improve gemcitabine sensitivity for at least some gemcitabine resistant cells regardless of CDA expression. Importantly, THU has been clinically available, has been established as safe, and is more reasonably priced than newly developed molecular targeted drugs. Further research is necessary to discover which cell types are appropriate for THU treatment to control proliferation. With improved THU stability in vivo, a combination treatment could be considered a more feasible and effective therapeutic approach than existing ones for advanced pancreatic carcinoma.
